# *In Vitro* analysis of the effect of mono-(2-ethylhexyl) phthalate (MEHP) exposure on macrophage inflammatory responses in relationship to Leydig cell steroid production

**DOI:** 10.3389/ftox.2025.1636395

**Published:** 2025-09-29

**Authors:** Akhil Adla, Allison Lunney, Barry Zirkin, Kassim Traore

**Affiliations:** ^1^ University of Tennessee Health Science Center, Memphis, TN, United States; ^2^ Duquesne University College of Osteopathic Medicine, Pittsburgh, PA, United States; ^3^ Johns Hopkins Bloomberg School of Public Health, Baltimore, MD, United States

**Keywords:** macrophage, Leydig cells, phthalate, mitochondria, TNF-α, progesterone

## Abstract

Macrophages, essential components of the innate immune system, are considered to be involved in the regulation of Leydig cell steroidogenesis, though by mechanisms that remain uncertain. Mono-(2-ethylhexyl) phthalate (MEHP), the active metabolite of di-(2-ethylhexyl) phthalate (DEHP), has been shown to affect testosterone production directly via its effects on Leydig cells, but also has been implicated in immune system modulation. These observations raise the possibility that MEHP might affect male steroidogenesis both by its direct effects on Leydig cells and perhaps also indirectly through its effects on macrophages. As yet, however, MEHP effects on macrophages and the potential relationship between macrophage response and Leydig cell steroidogenic function are poorly understood. Using *in vitro* methodology, we investigated the effects of MEHP on macrophage function and of downstream effects of changes in macrophage function on Leydig cell steroidogenesis. Mouse macrophage RAW 264.7 cells were cultured with MEHP (0–300 µM) for 24 h. Significant dose-dependent changes were seen in these cells in response to MEHP exposure, including increased cell size and granularity, increased mitochondrial content and membrane potential, decreased ATP production and oxygen consumption, and elevated intracellular and mitochondrial-derived oxidative stress. These changes suggested a pro-inflammatory response of the RAW 264.7 cells to MEHP. MEHP exposure activated the p38 MAPK pathway linking oxidative stress to inflammatory signaling and induced a dose-dependent increase in TNF-α secretion. *In vitro* exposure of MA-10 Leydig cells to TNF-α was found to inhibit steroid (progesterone) production by these cells. The observations, taken together, that TNF-α was secreted by MEHP-activated macrophages and that exposure to TNF-α can inhibit LH-stimulated steroid (progesterone) production by MA-10 Leydig cells suggest the possibility of the involvement of an immune-mediated mechanism resulting from MEHP exposure on impaired Leydig cell steroid production.

## Introduction

Di-(2-ethylhexyl) phthalate (DEHP), a widely used industrial plasticizer, is found in numerous consumer products, leading to widespread human exposure (Rowdhwal and Chen, 2018). DEHP and its bioactive metabolite, mono-(2-ethylhexyl) phthalate (MEHP), have been detected in human amniotic fluid, placenta, urine, blood, and saliva (Li et al., 2022a; Kessler et al., 2012; Martinez-Razo et al., 2021), raising concerns regarding their endocrine-disrupting potential and reproductive toxicity. Indeed, DEHP exposure has been shown to be associated with reduced testosterone production by the rat testis (Rowdhwal and Chen, 2018; Culty et al., 2008; Walker et al., 2021). As yet, however, the underlying mechanisms remain unclear.

Macrophages, essential components of the innate immune system, have been suggested to be involved in the regulation of testicular function, including Leydig cell steroidogenesis (Gu et al., 2022; Hales, 2002). The mechanisms underlying the functional relationship between macrophages and Leydig cells remain unclear. However, it has been suggested, though not proven, that macrophages might impact Leydig cells at least in part by regulating the intratesticular cytokine environment (Gu et al., 2022). In response to environmental stressors, macrophages can undergo metabolic reprogramming that influence their activation state and cytokine secretion profile (Gleeson and Sheedy, 2016; Wissfeld et al., 2022; Ganeshan and Chawla, 2014). Pro-inflammatory (M1) macrophages rely predominantly on glycolysis and generate reactive oxygen species (ROS), whereas anti-inflammatory (M2) macrophages favor oxidative phosphorylation (OXPHOS) and fatty acid oxidation (Martinez et al., 2008). Alterations in these metabolic pathways can shift macrophage polarization, thus stimulating inflammatory response and potentially impacting surrounding cells, including Leydig cells (Kelly and O'Neill, 2015; Viola et al., 2019).

MEHP has been reported to stimulate the release of tumor necrosis factor (TNF-α) and interleukin in RAW264.7 cells, a murine macrophage cell line derived from BALB/c mouse leukemia (Bolling et al., 2012; Park et al., 2019). DEHP has been shown to enhance IL-4 production in CD4^+^ T cells (Lee et al., 2004). The mechanism by which MEHP enhances cytokine production is not well characterized. However, it has been shown that mitogen-activated protein kinases (MAPKs) can modulate cytokine production, and that MEHP can induce phosphorylated MAPKs by many cell types (Qigen et al., 2023; Bolling et al., 2012). The three major MAPK families, the extracellular signal-regulated kinases (ERKs), the c-jun NH2 -terminal kinases (JNK), and the p38 MAPKs are protein kinases that require dual phosphorylation for activity (Roux and Blenis, 2004). In contrast to the ERKs, which are mainly activated by growth factors and other mitogenic stimuli, JNK and p38 MAPKs are activated in response to a number of stress stimuli (Roux and Blenis, 2004; Johnson and Lapadat, 2002). The latter, p38, is known to regulate the production of many pro-inflammatory macrophage cytokines that are associated with inflammation in various tissues (Schieven, 2005; Bachstetter and Van Eldik, 2010). Recently, phthalate exposure was reported to result in increased ROS production in many cell types including macrophages (Shi et al., 2023; Zhou et al., 2013; Traore et al., 2021; Zhou et al., 2019), suggesting that ROS formation may contribute to a pro-inflammatory response after MEHP exposure. Indeed, ROS has been shown to induce phosphorylation of p38 (Debattisti et al., 2017) and to increase inflammatory mediators such as tumor necrosis factor (TNF)-α (Blaser et al., 2016).

In the current studies, we used RAW 264.7 cells to first examine MEHP-induced changes in cytokine secretion, mitochondrial function, and ROS generation. We then addressed the effect of macrophage-derived inflammatory mediator tumor necrosis factor-alpha (TNF-α), produced by the RAW264.7 cells in response to MEHP, on MA10 Leydig cell steroidogenesis. Our findings indicate that MEHP exposure of RAW264.7 cells can disrupt mitochondrial homeostasis and promote activation, and that the stimulation of TNF-α production by these cells can affect steroid production by MA-10 Leydig cells. These results, together with previously published studies (Jones et al., 1993; Traore and Zirkin, 2024), suggest an immune-metabolic mechanism through which environmental toxicants may impair endocrine function indirectly through effects on macrophages as well through direct effects on the Leydig cells themselves.

## Materials and methods

### Reagents

MEHP was purchased from Sigma-Aldrich (St. Louis, MO). The JC-1 dye (Mitochondrial Membrane Potential Probe) was obtained from Molecular Probes™ Invitrogen. 2′,7′-Dichlorofluorescein diacetate (DCFH-DA) was obtained from Molecular Probes™ Invitrogen and from Sigma- Aldrich. All antibodies used for Western blots were purchased from Cell Signaling Technology Inc. (Danvers, MA). Cell viability was assessed using the MTT [3-(4,5-dimethylthiazol-2-yl)-2,5-diphenyltetrazolium bromide] assay following the manufacturer’s instructions (Trevigen, Gaithersburg, MD). Absorbance was assessed using the EL 340-microplate reader at 550–600 nm wavelengths. The Seahorse XFp Real-Time ATP Rate Assay kits were purchased from Agilent Technologies, Inc.

### Cell culture and treatments

RAW 264.7 cells were obtained from the American Type Culture Collection (ATCC, Rockville, MD). Cells were cultured in RPMI medium supplemented with 10% fetal bovine serum (FBS) and 1% penicillin/streptomycin. Cultures were maintained in a humidified incubator at 37 °C with 5% CO_2_ and 95% humidity. Cells were passaged upon reaching 85%–90% confluence, detached using trypsin, and subcultured in T-75 flasks. For experimental treatments, cells were exposed to MEHP (10, 100, 200, or 300 μM) or cultured with vehicle (DMSO) at a maximal final concentration of 0.05%.

The concentration of 10 μM was chosen as it reflects human blood levels (Li et al., 2022b), while 100 μM represents potential cumulative exposure to multiple phthalates, as previously described (Howdeshell et al., 2017). The higher concentrations (200 and 300 μM) were included to explore dose-dependent effects and to assess potential cytotoxic thresholds, as reported in prior toxicological studies (Li et al., 2022b).

MA-10 mouse Leydig tumor cells were generously provided by Dr. Mario Ascoli (University of Iowa). The cells were cultured at 34 °C in DMEM supplemented with 10% horse serum, 5% FBS, 4.76 mg/mL HEPES, 1.2 mg/mL sodium bicarbonate, and 25 μg/mL gentamicin. Unlike primary Leydig cells, MA-10 cells mainly convert cholesterol to progesterone, not testosterone, due to limited expression of 17α-hydroxylase/17,20 lyase (CYP17A1).

### TNF-α production

RAW 264.7 cells were incubated in culture medium containing MEHP (10–300 µM) for 24 h. Control cells, were incubated with vehicle alone. TNF-α production in the supernatant was quantified by enzyme-linked immunosorbent assay (ELISA) using the R&D Systems kit (Catalog No. MTA00B, R&D System), with each sample measured in triplicate. TNF-α levels were normalized to total protein levels per flask. To minimize the risk of endotoxin contamination, we used endotoxin-free reagents and materials throughout our experiments, including sterile techniques and endotoxin-free water and plasticware.

### Effect of TNF-α on progesterone production

The effect of TNF-α on LH-stimulated progesterone production was assessed by seeding 1 × 10^6^ MA-10 cells into 10 mL culture flasks overnight. To assess the acute inhibitory effects of TNF-α on MA-10 Leydig cell steroidogenic function, as previously described (Budnik et al., 1999), cells were pre-incubated with or without TNF-α (1 or 10 ng/mL) for 6 h and then incubated in fresh culture medium containing LH (100 ng/mL) for 2 h. Progesterone production in the supernatant was quantified by enzyme-linked immunosorbent assay (ELISA) using the Enzo Life Sciences kit (Catalog No. ADI-901-011), with each sample measured in triplicate. Progesterone levels were normalized to total protein levels per flask (Hauet et al., 2005).

### Cell viability

The effect of TNF-α (1 and 10 ng/mL) and MEHP (10–300 µM) exposures on cell viability was assessed with the 3-(4,5-dimethylthiazol-2-yl)-2,5-diphenyl tetrazolium bromide (MTT) cell viability/cytotoxicity assay (Benov, 2021).

### Immunoblot analysis

For immunoblotting to assess STAR levels in MA-10 cells, phospho-p38 in RAW 264.7 cells, and tubulin in both cell types, three million cells were seeded per condition and allowed to adhere overnight. Cells were then incubated with MEHP (10–300 μM) or TNF-α (1 or 10 ng/mL) for the indicated times. After incubation, cells were rinsed twice with cold phosphate-buffered saline (PBS) and lysed in lysis buffer (Tris-HCL pH 7.4, 50 mM, NaCl 150 mM, Triton x 100 1%, and EDTA 5 mM). Lysates were centrifuged at 13,000 g for 10 min. Samples, each containing 30 μg of protein in loading buffer, were separated by 10% SDS-polyacrylamide gel electrophoresis. The separated proteins were transferred to a polyvinylidene fluoride membrane (Novex, Life Technologies, MA). The membrane was blocked by incubating in PBS containing 0.01% Tween-20 (PBST) and 2.5% BSA overnight at 4 °C. Immunoblotting was performed using rabbit antibodies against mouse STAR protein (Catalog No. 8449S, Cell Signaling Technology, MA) in MA10 cells or against phospho-p38 in RAW 264.7 cells (Catalog No. 9211s, Cell Signaling Technology, MA) at 1:1,000 dilution for 1 h. After washing the blots, alkaline phosphatase-conjugated goat anti-rabbit IgG, diluted 1:2,000, was added, and the blots were incubated for 30 min. The membrane was washed with PBST, and bound antibodies were visualized by adding chromogen 5-bromo-4-chloro-3-indolyl phosphate/nitroblue tetrazolium (BCIP/NBT) substrate (Life Sciences, MA).

### Hydrogen peroxide (H_2_O_2_) generation

The cellular redox status was assessed by measuring hydrogen peroxide (H_2_O_2_) generation using the fluorogenic, cell-permeant dye 2′,7′-dichlorofluorescin diacetate (DCFH-DA), as previously described (Rothe and Valet, 1990). RAW 264.7 cells were treated with vehicle alone (as described above) or MEHP (10–300 μM) for 24 h, followed by incubation with 10 μM DCFH-DA for 30 min. After harvesting, cells were washed twice with PBS, and the fluorescence intensity of 10,000 cells was quantified using fluorescence-activated cell sorting on a Guava^®^ easyCyte HT flow cytometer. Fluorescence data were analyzed using GuavaSoft^®^ software (InCyte module, Guava^®^ easyCyte HT Systems).

### Superoxide generation

Mitochondrial superoxide was detected using MitoSOX™ Red reagent. RAW 264.7 cells were treated with vehicle alone (as described above) or MEHP (10–300 μM) for 24 h, followed by incubation with 2 μM MitoSOX™ in PBS for 10 min. The fluorescence intensity of 10,000 cells was quantified using a Guava^®^ easyCyte HT flow cytometer, and data were analyzed with GuavaSoft^®^ software (InCyte module, Guava^®^ easyCyte HT Systems).

### Basal oxygen consumption rate (OCR) and real-time ATP rate measurement

Basal oxygen consumption rate (OCR) and real-time ATP production were assessed using the Agilent Seahorse XFp Real-Time ATP Rate Assay (Catalog No. 103591-100, Agilent Technologies), which quantifies total ATP production in live cells and distinguishes ATP derived from mitochondrial oxidative phosphorylation versus glycolysis. Basal OCR and extracellular acidification rate (ECAR) were measured. Metabolic modulators oligomycin, and a combination of rotenone and antimycin A were sequentially injected to calculate mitochondrial and glycolytic ATP production rates. Oligomycin inhibits mitochondrial ATP synthase, causing a decrease in OCR that enables quantification of mitochondrial ATP production. ECAR data were used to calculate the total proton efflux rate (PER). Complete inhibition of mitochondrial respiration by rotenone plus antimycin A causes mitochondria-associated acidification, which, combined with PER data, allows calculation of glycolytic ATP production rates.

### Mitochondrial membrane potential (ΔΨm)

Mitochondrial membrane potential (ΔΨm) was assessed using the cationic, potentiometric dye JC-1 (Thermo Fisher Scientific). RAW 264.7 cells were cultured in RPMI medium to ∼75% confluency (∼2 × 10^6^ cells) in T25 flasks (Fisher Scientific). Cells were treated with MEHP (10–300 μM) for 24 h at 37 °C and 5% CO_2_ in triplicate. After treatment, cells were harvested and resuspended in 1 mL PBS containing 2.5 μM JC-1, followed by incubation for 20 min at 37 °C. Cells were washed with PBS to remove excess dye and resuspended in 500 μL of 0.1% PBS/BSA for flow cytometric analysis. JC-1 fluorescence was measured using a Guava^®^ easyCyte HT flow cytometer (Guava^®^ easyCyte HT Systems), and data were analyzed using GuavaSoft^®^ software (MitoPotential module, Luminex Corporation). JC-1 monomer (green) fluorescence was detected at 529 nm following excitation at 514 nm. Mitochondrial membrane potential (ΔΨm) was assessed by calculating the ratio of cells with high membrane potential (purple-labeled) to those with low membrane potential (blue-labeled). A higher ratio indicates increased mitochondrial polarization.

### Statistical analysis

Data presented are expressed as mean ± standard error of three replicate samples from three different experiments. Group means were evaluated by one-way ANOVA. The Tukey-Kramer HSD test was performed using JMP software (SAS, Statistical Discovery, and NC) to determine significant differences. Results were considered significant at P < 0.05.

## Results

The following studies were designed to determine the molecular mechanisms by which MEHP affects macrophage function, and how changes in macrophage function might affect MA10 Leydig cell steroidogenesis.

### Effects of MEHP on RAW 264.7 macrophage cell morphology

**FIGURE 1:**
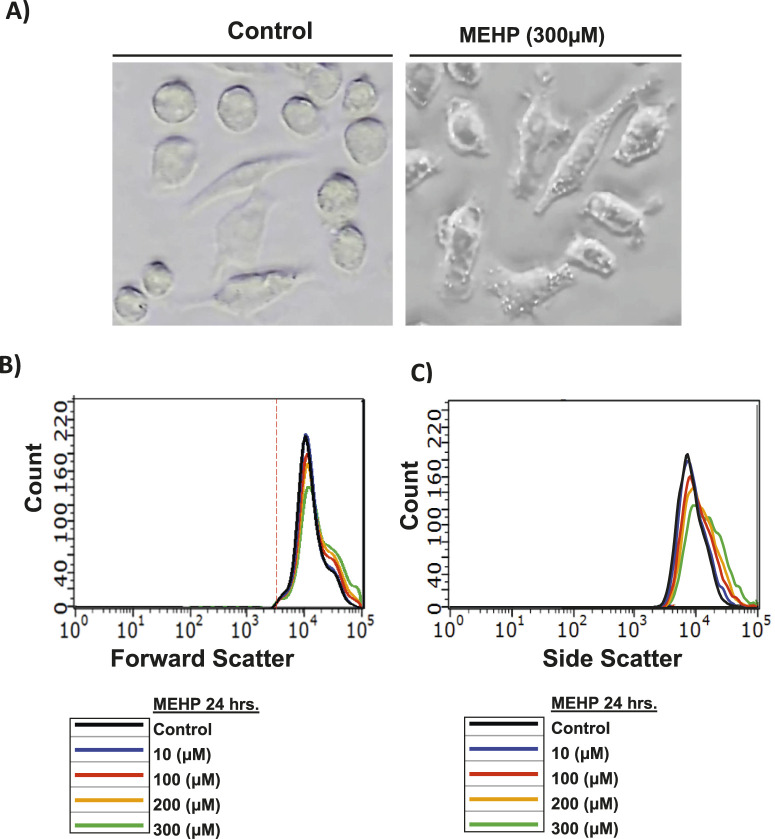
**(A)** Morphological changes in RAW 264.7 cells after their incubation with MEHP (300 µM) compared to control. **(B,C)** Flow cytometry analysis was performed to evaluate changes in cell morphology. Forward scatter (FSC) was used to assess cell size **(B)**, while side scatter (SSC) measured intracellular granularity and complexity **(C)**.

Mouse macrophage RAW 264.7 cells were treated with vehicle alone or with MEHP (10–300 µM) for 24 h. As seen in Figure 1A, light microscopy revealed morphological differences between control and MEHP-treated cells. Flow cytometry analysis data showed a dose-dependent increase in forward scatter (FSC), indicating cell enlargement (Figure 1B). Side scatter (SSC) was also increased with MEHP treatment, reflecting greater intracellular granularity and complexity (Figure 1C).

### MEHP induces ROS generation in RAW 264.7 cells

**FIGURE 2:**
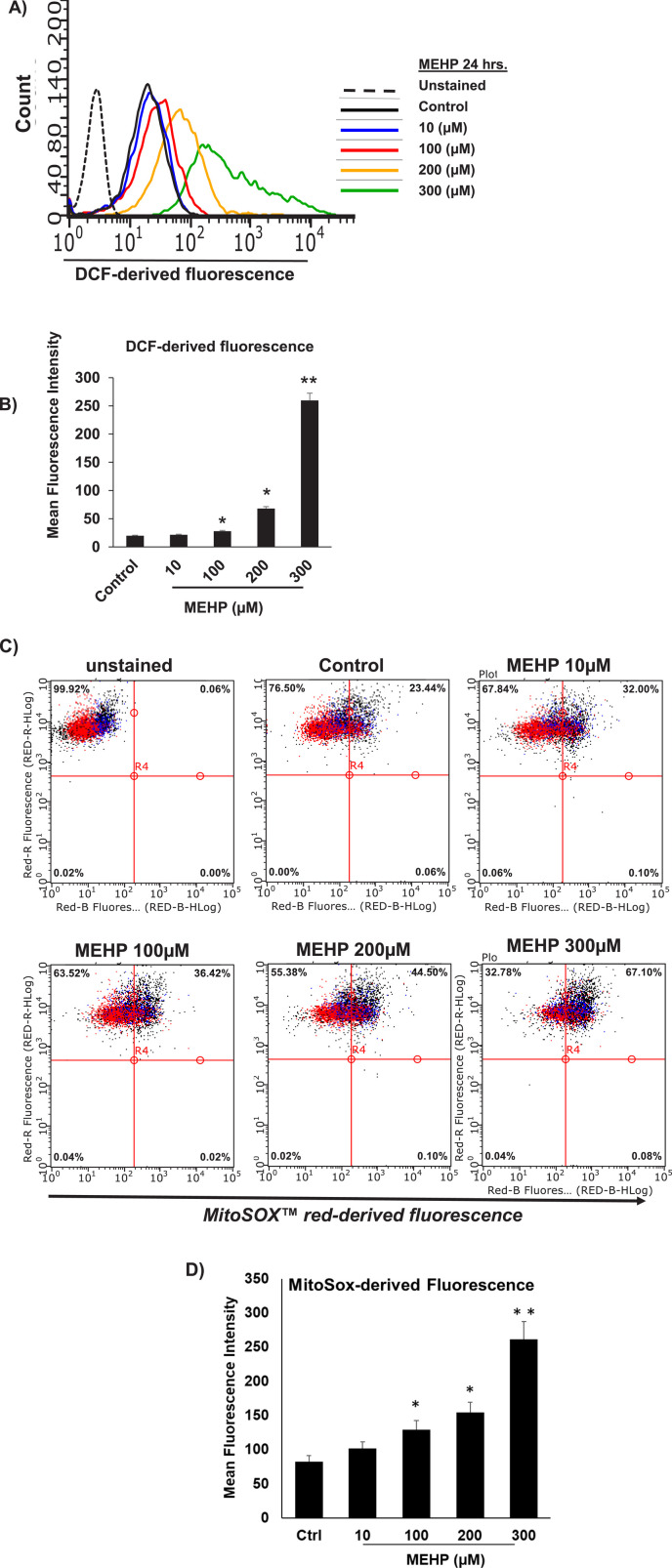
MEHP effects on RAW 264.7 cell reactive oxygen species (ROS) generation. Cells were incubated for 24 h in medium containing either vehicle alone or increasing concentrations of MEHP (10–300 μM). After incubation, cells were analyzed for ROS generation using DCFH-DA (2′,7′-dichlorodihydrofluorescein diacetate). Unstained are cells without DCFH-DA, used to determine background fluorescence. **(A)** Histogram showing flow cytometry analysis of DCFH-DA fluorescence in cells across treatment groups. **(B)** Bar graph showing the mean DCF-derived fluorescence intensity (MFI) for each treatment group. Superoxide generation by mitochondria of MEHP-incubated RAW 264.7 cells was evaluated using flow cytometry analysis of MitoSOX Red-derived fluorescence. **(C)** Dot plot showing flow cytometry analysis of MitoSOX Red fluorescence in cells across treatment groups. Unstained are cells without MitoSOX Red, used to determine background fluorescence. **(D)** Bar graph showing the mean fluorescence intensity (MFI) for each treatment group. Mean ± SEM from three independent experiments. *P < 0.05, **P < 0.01.

Reactive oxygen species (ROS) generation by macrophages has been linked to the inflammatory response of these cells (Rendra et al., 2019; Canton et al., 2021). To determine whether the response of macrophages to MEHP might involve the generation of intracellular ROS, RAW 264.7 cells were treated with increasing doses of MEHP and ROS production was measured using 2′,7′- dichlorofluorescein (DCF), a dye that fluoresces upon oxidation by hydrogen peroxide (H_2_O_2_). Cells were incubated for 24 h with 0–300 μM MEHP and then DCFH-DA (2′,7′-dichlorofluorescein diacetate) for 30 min, and analyzed by flow cytometry. MEHP treatment caused dose-dependent increases in DCF fluorescence, indicated by a rightward shift in the curve (Figure 2A). Mean cellular fluorescence also increased in response to MEHP exposure (Figure 2B).

We further investigated the impact of MEHP on intra-mitochondrial superoxide production using flow cytometry analysis of MitoSox Red-derived fluorescence. MitoSox red is a dye that selectively detects superoxide anions in the mitochondria. As shown in Figure 2C, MEHP exposure led to a dose-dependent shift in cell distribution from low fluorescence (upper-left quadrant) to high fluorescence (upper-right quadrant), indicating an increase in superoxide-producing cells. Higher MEHP concentrations resulted in a greater number of MitoSox Red-positive cells, with a significant increase in MitoSox-derived fluorescence per cell at 100, 200, and 300 μM MEHP (Figure 2D).

### MEHP-induced ROS signals MAPK-p38 activation

**FIGURE 3:**
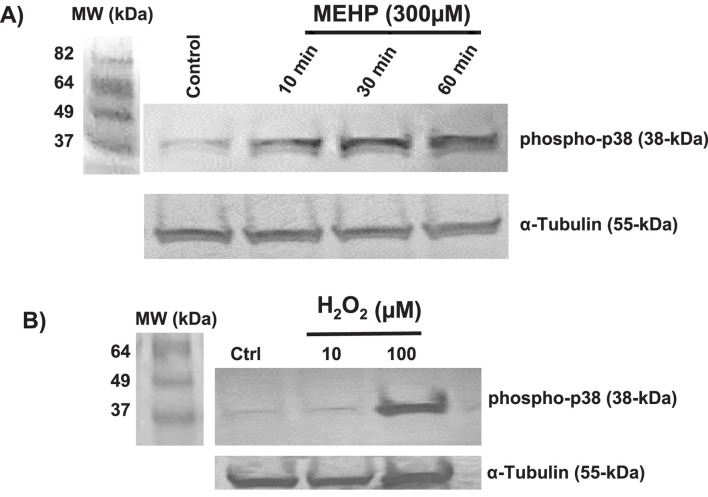
**(A)** Time-dependent effects of MEHP (300 μM) on MAPK p38 phosphorylation, as assessed by Western blot using a rabbit anti-mouse phospho-p38 antibody. **(B)** Effects of 30-min incubation with increasing 10 and 100 μM concentrations of hydrogen peroxide (H_2_O_2_) on phospho-p38 levels.

Mitogen-activated protein kinases (MAPKs) play crucial roles in modulating inflammatory responses (Chen et al., 2023; Arthur and Ley, 2013). MAPK- p38 activation plays a key role in M1 macrophage polarization, drives pro-inflammatory responses, and promotes production of IL-1β, TNF-α, and IL-6 (Arthur and Ley, 2013). Previous studies have shown that MEHP can activate the p38 MAPK signaling pathway in mouse macrophage RAW 264.7 cells, leading to increased production of pro-inflammatory cytokines (Bolling et al., 2012) ROS generation has been linked to p38 activation in response to hypoxia in cardiomyocytes (Kulisz et al., 2002). We sought to determine whether there might be a direct link between MEHP exposure-induced ROS and MAPK-p38 activation in RAW 264.7 cells. As shown in Figure 3A, basal phospho-p38 levels were low in control cells but increased significantly in time-dependent fashion, with the highest levels recorded at 30 min of exposure to MEHP (300 μM). This accumulation of phospho-p38 correlated with increasing accumulation of ROS (Figures 2A,B). Treatment with H_2_O_2_ (10 and 100 µM) mimicked the effects of MEHP, inducing phospho-p38 accumulation and further confirming the direct relationship between ROS production and p38 phosphorylation in RAW 264.7 cells (Figure 3B).

### MEHP disrupts mitochondrial function and reduces oxidative metabolism in macrophages

**FIGURE 4:**
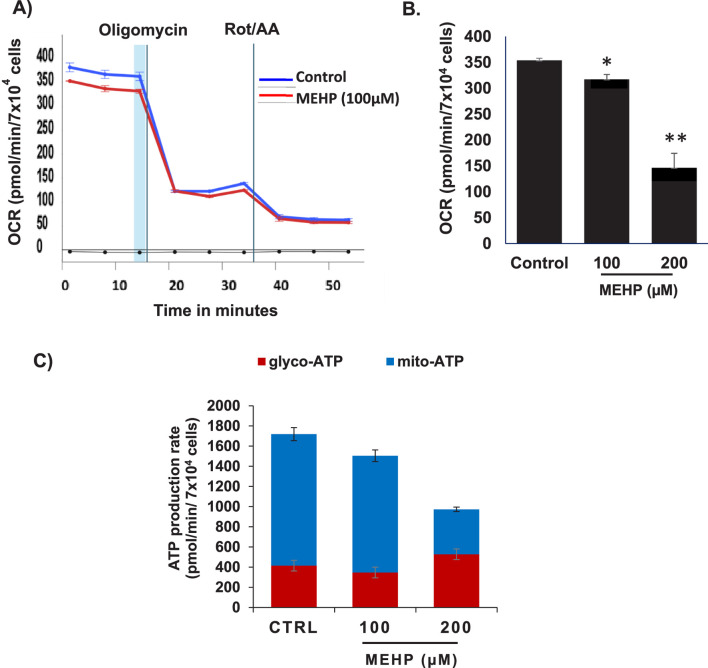
Effects of MEHP on basal oxygen consumption rate (OCR) in RAW 264.7 cells were assessed using the Agilent Seahorse XFp Real-Time ATP Rate Assay to evaluate its impact on macrophage ATP production. **(A)** Real-time OCR measurements are shown in blue for untreated control cells and in red for cells treated with MEHP (100 µM). Oligomycin, an ATP synthase inhibitor, was used to reduce OCR, enabling calculation of mitochondrial ATP production. Subsequent addition of rotenone and antimycin A (Rot/AA) inhibited mitochondrial respiration, allowing assessment of glycolytic ATP production. **(B)** Dose-dependent effects of MEHP on OCR. **(C)** Dose-dependent effects of MEHP on ATP production rates. Mean ± SEM from three independent experiments. *P < 0.05, **P < 0.01.

Mitochondrial metabolism is essential for macrophage activation and the regulation of inflammatory responses (Wang et al., 2021; Qing et al., 2020). Recent studies emphasize metabolic reprogramming, particularly changes in oxidative phosphorylation (OXPHOS), as a key driver of macrophage polarization (Wang et al., 2021). To assess how MEHP affects macrophage metabolism, we used the Agilent Seahorse XFp Real-Time ATP Rate Assay to profile RAW 264.7 cells. This assay quantifies total ATP production in live cells, distinguishing between mitochondrial (OXPHOS-derived) and glycolytic ATP contributions. Basal oxygen consumption rate (OCR) and extracellular acidification rate (ECAR) were measured, followed by sequential addition of metabolic inhibitors. Oligomycin, an ATP synthase inhibitor, reduces OCR, enabling calculation of mitochondrial ATP production. Subsequent treatment with rotenone and antimycin A fully inhibits mitochondrial respiration, allowing assessment of glycolytic ATP production based on mitochondrial-associated acidification and proton efflux rate (PER). MEHP exposure significantly reduced OCR (Figures 4A,B), and mitochondrial ATP production was markedly decreased (Figure 4C), indicating mitochondrial dysfunction.

### MEHP exposure increases in the intracellular granularity and the mitochondrial count

**FIGURE 5:**
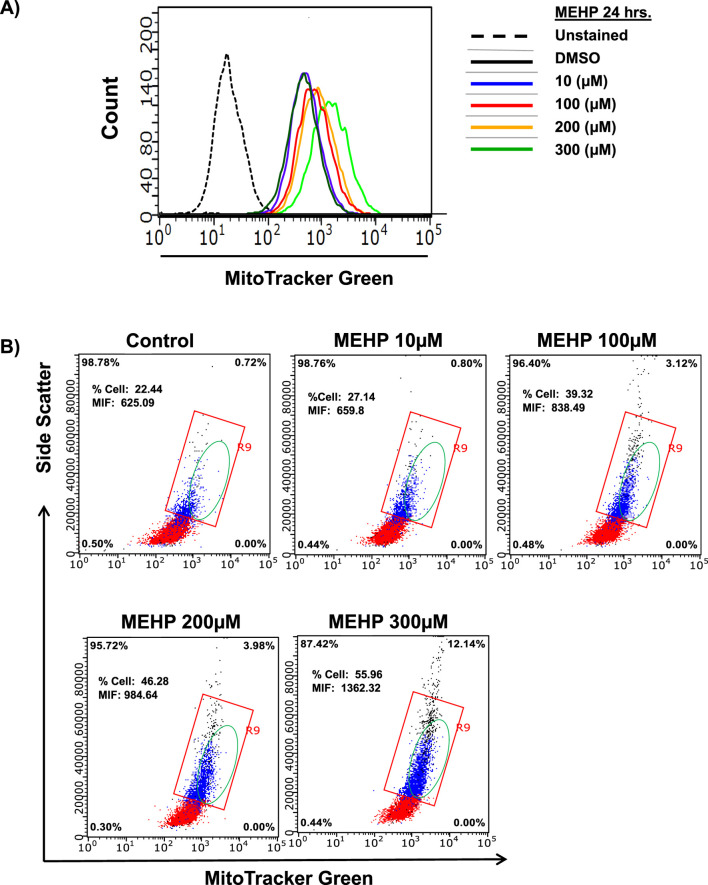
Effects of MEHP (10–300 μM, 24 h) on mitochondrial mass in RAW 264.7 cells, assessed by flow cytometry using MitoTracker Green-derived fluorescence. Unstained are cells without MitoTracker Green, used to determine background fluorescence. **(A)** Representative histogram showing a dose-dependent rightward shift in MitoTracker Green fluorescence, indicating an increased population of cells with elevated mitochondrial mass. **(B)** Intracellular granularity and complexity in response to increasing MEHP concentrations, measured by side scatter (SSC). The red square highlights a cell population with relatively high side scatter (SSC), indicating increased granularity. The green-circled population represents cells exhibiting both elevated MitoTracker Green fluorescence and increased SSC, suggesting enhanced mitochondrial content and greater cellular complexity.

An increase in mitochondrial mass is essential to meet the elevated energy demands and reactive oxygen species (ROS) production associated with macrophage activation and inflammatory signaling (Jones and Divakaruni, 2020; Wang et al., 2021). To assess mitochondrial mass, we performed flow cytometry analysis using MitoTracker Green fluorescence, which reflects total mitochondrial mass, alongside measurements of side scatter to evaluate intracellular granularity and complexity within the same cell population. MEHP exposure led to a dose-dependent increase in MitoTracker Green fluorescence (Figure 5A), indicating elevated mitochondrial mass. This increase was accompanied by a corresponding rise in side scatter values, suggesting enhanced intracellular granularity and complexity (Figure 5B).

### MEHP disrupts mitochondrial ATP production and promotes increased mitochondrial membrane potential (ΔΨm)

**FIGURE 6:**
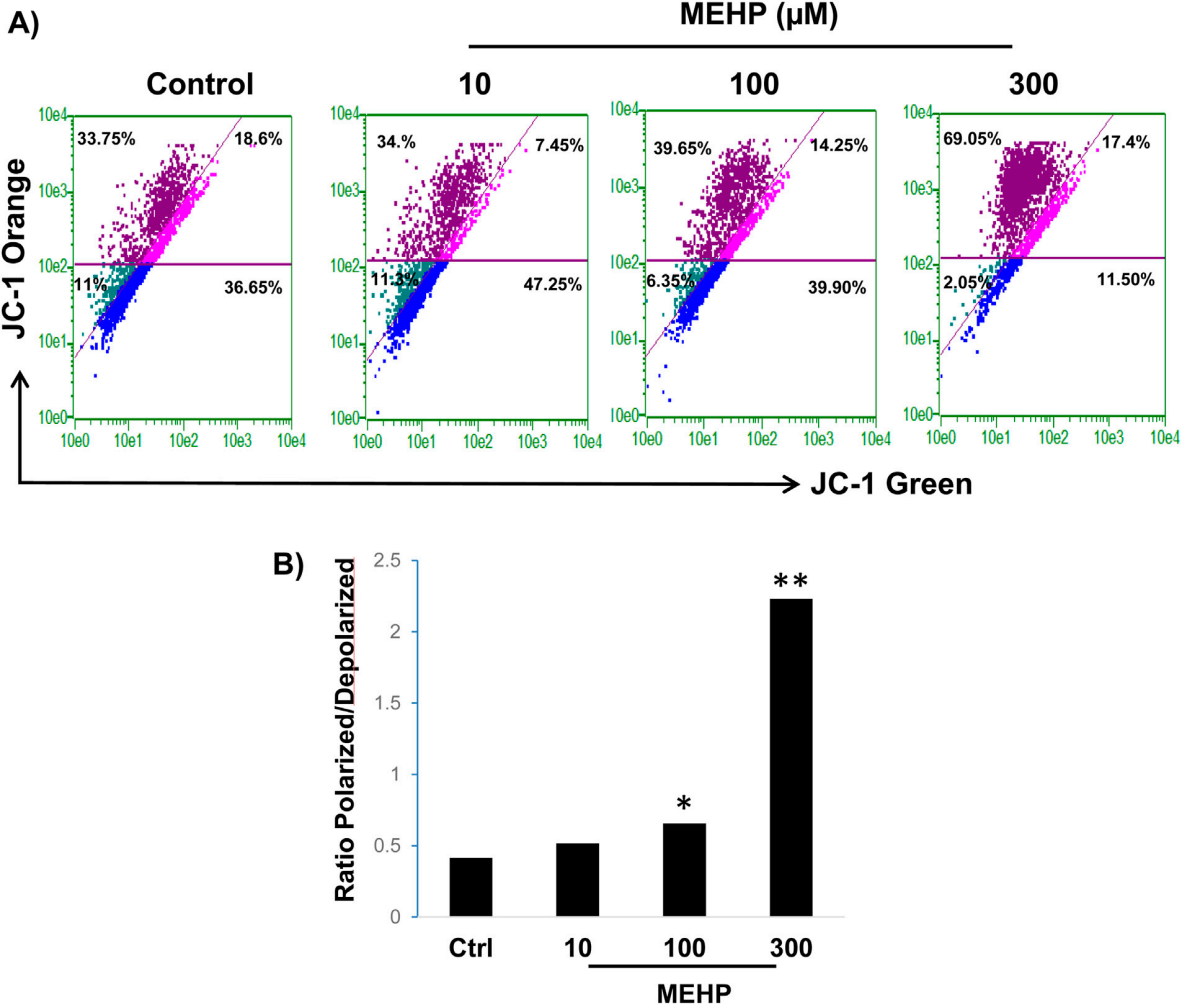
Effects of MEHP on mitochondrial membrane potential in RAW 264.7 cells. RAW 264.7 cells (1 × 10^6^) were treated with MEHP (10–300 μM) for 24 h, and mitochondrial membrane potential was measured using JC-1 dye and flow cytometry. JC-1 accumulates in mitochondria based on membrane potential, emitting green fluorescence (∼529 nm) at low potential (monomer form) and red fluorescence (∼590 nm) at high potential (J-aggregates). **(A)** Shift of MEHP-treated cells into the upper left quadrant (dark purple) represents cells with high mitochondrial membrane potential (polarized cells), indicating an increase in mitochondrial polarization in response to MEHP exposure. **(B)** Ratio of polarized to depolarized cells in response to MEHP *P < 0.05, **P < 0.01.

Cells that rely primarily on glycolysis for ATP production, such as those activated by MEHP, are expected to exhibit elevated mitochondrial membrane potential (ΔΨm) (Zorova et al., 2018). This increase occurs because the proton gradient generated by complexes I, III, and IV is not consumed by oxidative phosphorylation, resulting in its accumulation. We used JC-1 dye in conjunction with flow cytometry to assess the impact of MEHP on mitochondrial membrane potential (ΔΨm). JC-1 (5,5′,6,6′-tetrachloro-1,1′,3,3′-tetraethylbenzimidazolylcarbocyanine iodide) is a cationic, lipophilic dye widely utilized to evaluate ΔΨm in live cells, as it accumulates within mitochondria in a potential-dependent manner. In cells with low mitochondrial membrane potential (ΔΨm), JC-1 remains in its monomeric form and emits green fluorescence (∼529 nm). In cells with high ΔΨm, JC-1 accumulates in the mitochondria and forms J-aggregates, emitting red fluorescence (∼590 nm). The ratio of red to green fluorescence serves as an indicator of mitochondrial polarization status. We detected a dose-dependent rise in ΔΨm following MEHP exposure. This was indicated by a shift in cell populations from the lower right quadrant (low ΔΨm) to the upper left quadrant (high ΔΨm) in flow cytometry plots (Figure 6A). Correspondingly, the ratio of polarized to depolarized cells increased with MEHP treatment (Figure 6B).

### MEHP induces mitochondrial biogenesis in RAW 264.7 cells

**FIGURE 7:**
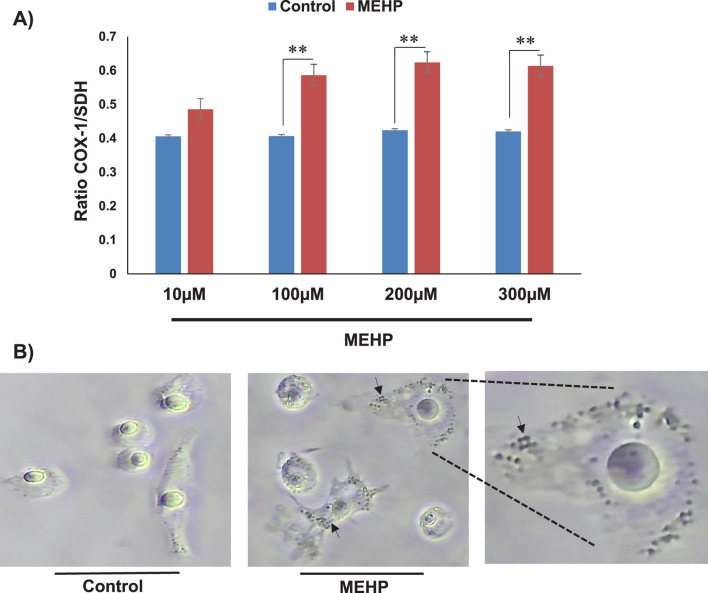
Mitochondrial biogenesis following MEHP exposure assessed by In-Cell ELISA and immunocytochemistry. **(A)** Quantitative analysis revealed a higher COX-1/SDH-A ratio in MEHP-treated cells. **(B)** Light microscopy images of stained cells, revealing increased mitochondrial content in response to MEHP. The arrows show dividing mitochondria in MEHP-treated cells.

To determine whether the MEHP-induced increase in mitochondrial mass is attributable to enhanced mitochondrial biogenesis, we performed an In-Cell ELISA MitoBiogenesis assay (Cat. NC0292957). Cells were permeabilized and sequentially stained with antibodies against cytochrome c oxidase subunit I (COX-1), a mitochondrial-encoded protein, and succinate dehydrogenase subunit A (SDH-A), a nuclear-encoded mitochondrial protein. Quantitative analysis revealed an increased COX-1/SDH-A ratio following MEHP treatment, suggesting a potential upregulation of mitochondrial biogenesis (Figure 7A). This finding was further supported by light microscopy images of COX-1 and SDH-stained cells, which showed increased mitochondrial content in MEHP-treated cells (Figure 7B).

### MEHP effect on macrophage RAW 264.7 cell TNF-α cytokine production and of TNF-α effect on steroid biosynthesis

**FIGURE 8:**
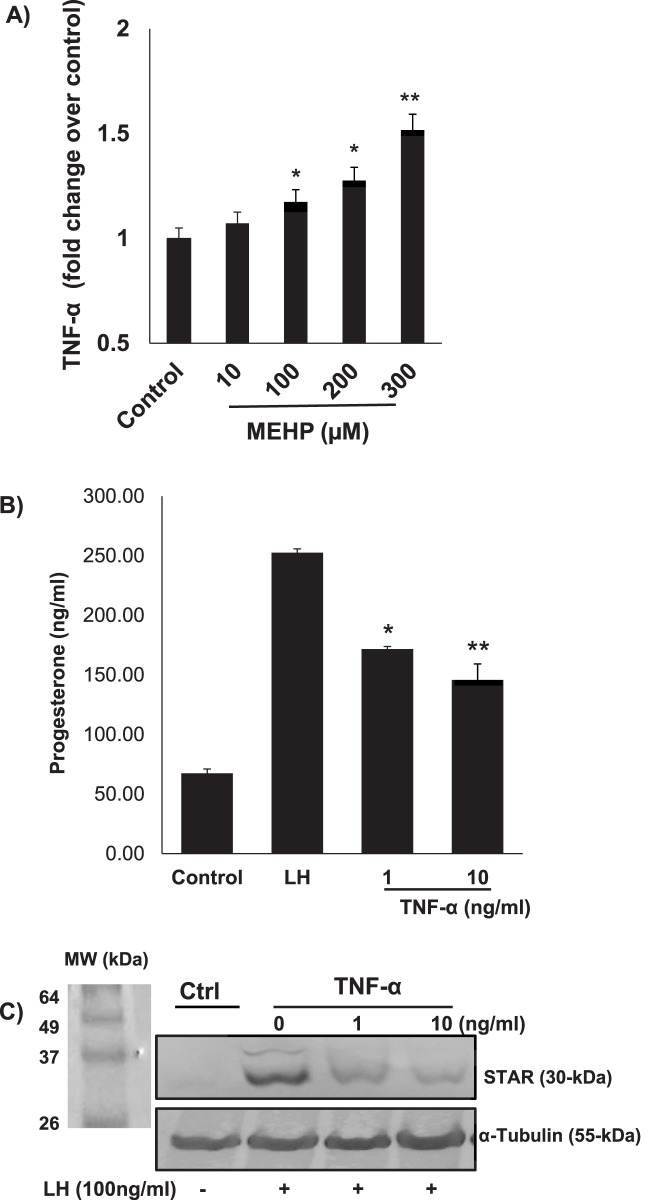
**(A)** Pro-inflammatory effect of MEHP on RAW 264.7 mouse macrophages. Cells were incubated with vehicle alone or with increasing concentrations of MEHP (10–300 μM) for 24 h. TNF-α was quantified using an enzyme-linked immunosorbent assay (ELISA). Data shown are mean ± SEM of three separate experiments. **(B)** Effect of TNF-α on the steroidogenic function of LH-stimulated MA10 Leydig cells. Significantly different from control (basal): *P < 0.05, **P < 0.01. **(C)** TNF-α effect on 30 kDa STAR protein, assessed by immunoblot analysis of RAW 264.7 cell total proteins using rabbit anti-mouse STAR antibodies.

*In vitro* and *in vivo* phthalate exposures have been shown to result in reduced testosterone production by Leydig cells (Jones et al., 1993; Traore and Zirkin, 2024). We hypothesized that phthalate effects on steroidogenesis might involve not only direct effects on the steroid-producing Leydig cells but also indirect effects resulting from effects on the Leydig cell-associated macrophages. To begin to address this, we first incubated RAW 264.7 cells in medium containing MEHP (0–300 µM) for 24 h. Using an enzyme-linked immunosorbent assay (ELISA), we found that TNF- α, a cytokine known to play an important role in inflammation, was produced by the RAW 264.7 cells in response to MEHP and to increase significantly in response to increasing MEHP doses from 100 to 300 µM (Figure 8A). To evaluate the possible impact on steroid biosynthesis of the TNF-α induced by MEHP, we determined the effect of TNF-α on LH-induced steroidogenesis by MA-10 Leydig cells. MA-10 cells were pretreated with TNF-α (1 or 10 ng/mL) or PBS alone for 6 h, after which the cells were stimulated with LH for 2 h. The pretreatment of MA-10 cells with TNF-α for 6 h was based on prior reports demonstrating that this time frame is sufficient to induce a stable inflammatory response without causing excessive cytotoxicity (Budnik et al., 1999). The cells then were exposed to hCG for 2 h to mimic acute gonadotropin stimulation of androgen production, as previously described in many *in vitro* steroidogenesis assays. TNF-α exposure resulted in concentration-dependent reductions in LH-stimulated progesterone production at 1 and 10 ng/mL (Figure 8B). Consistent with reductions in steroid production, the expression levels of steroidogenic acute regulatory protein (STAR), a key transport protein in the steroidogenic process, were significantly reduced in response to TNF-α exposure (Figure 8C). None of the TNF concentrations affected cell viability, as determined by the MTT assay (data not shown).

## Discussion

Previous studies have shown that *in vivo* exposure to mono-(2-ethylhexyl) phthalate (MEHP), the active metabolite of di-(2-ethylhexyl) phthalate (DEHP), can affect the immune system (Li et al., 2025; Linghu et al., 2024), and have suggested that under inflammatory conditions macrophages might produce factors able to affect Leydig cell steroidogenesis (Hales, 2002). Such studies, taken together, raise the possibility that MEHP exposure might affect male steroidogenesis not only by direct effects on Leydig cells, which has been shown (Traore and Zirkin, 2024; Zhao et al., 2012), but also indirectly at least in part via effects on the macrophages with which the Leydig cells are associated in the testis.

The *in vitro* studies presented herein were designed to begin to address the possibility that MEHP exposure of RAW 264.7 cells in fact can result in their production of factors able to suppress Leydig cell steroidogenesis. Depending upon its dose, MEHP exposure of the RAW 264.7 cells resulted in increased side-scatter suggesting increased intracellular granularity, a hallmark of macrophage activation. This morphological change is consistent with the increased mitochondrial mass and membrane potential detected in MEHP-treated RAW 264.7 cells, suggesting mitochondrial reprogramming during the activation process. Macrophage activation is known to be driven by metabolic shifts through which pro-inflammatory (M1) macrophages exhibit increased glycolytic activity and ROS generation, while the anti-inflammatory (M2) macrophages rely mainly on the oxidative phosphorylation in the mitochondria (Viola et al., 2019; Kolliniati et al., 2022; Gupta and Sarangi, 2023; Mills et al., 2016). Our results suggest that MEHP exposure directs macrophages towards a pro-inflammatory state, as indicated by increased ROS generation and TNF-α production.

MEHP-induced ROS generation is a key finding in this study, as oxidative stress is a major driver of inflammatory responses (Mittal et al., 2014). The dose-dependent increase in DCF fluorescence and MitoSox Red-derived fluorescent cells confirms elevated intracellular and mitochondrial ROS production. This increased generation of ROS likely contributes to the activation of the p38 MAPK pathway, as demonstrated by increased phospho-p38 levels in MEHP treated cells (Tiwary and Richburg, 2023). The ROS-mediated activation of p38 MAPK has been well-documented in inflammatory responses (Averill-Bates, 2024), suggesting that MEHP exposure triggers a cascade of events leading to macrophage activation and cytokine secretion.

Our mitochondrial analysis data highlight potential mechanisms by which MEHP might induce macrophage activation. The increased MitoTracker Green-derived fluorescence and JC-1 staining suggest mitochondrial remodeling in response to MEHP, possibly as a compensatory response to oxidative stress. While mitochondrial biogenesis is generally associated with increased cellular function, the simultaneous mitochondrial dysfunction observed through reduced ATP production and OCR measurements indicates metabolic stress. These mitochondrial alterations most likely contribute to the inflammatory phenotype observed in MEHP-treated macrophages. The activation of macrophages, characterized by increased mitochondrial ROS generation, p38 MAPK activation, and TNF-α production, apparently establishes an inflammatory microenvironment able to result in suppression of Leydig cell steroidogenesis. Together, current and preceding findings highlight the potential endocrine-disrupting effects of phthalates beyond direct cytotoxicity, and emphasize the need for further cause-effect investigations into immune-metabolic interactions in reproductive toxicity.

Having demonstrated these effects of MEHP on macrophages, and previous observations that MEHP exposure *in vivo* and *in vitro* can affect Leydig cell steroidogenesis (Svechnikov et al., 2008; Zhao et al., 2012; Traore et al., 2021; Yang et al., 2025; Sekaran and Jagadeesan, 2015), a major objective of the current study was to assess whether MEHP-induced macrophage activation can affect MA10 Leydig cell function. Our results show that TNF-α, a pro-inflammatory cytokine, was produced by macrophages in response to MEHP, and that exposure of MA10 Leydig cells to TNF-α can inhibit LH-stimulated progesterone biosynthesis by MA10 Leydig cells. These findings suggest the possibility that MEHP’s detrimental effects on steroidogenesis may result not only from direct toxicity to Leydig cells, as shown previously (Leisegang and Henkel, 2018), but also indirectly through an immune-mediated mechanism. It should be noted, however, that other testicular cell types besides macrophages, particularly Sertoli cells, might also be direct targets of MEHP. Sertoli cells have been reported to produce TNF-α, and there are MEHP-induced adverse effects on Sertoli cell function (Yao et al., 2007; Chen et al., 2013; Yao et al., 2009). Thus, while our findings suggest MEHP-mediated induction of TNF-α in macrophages as a potential mechanism for impaired Leydig cell steroidogenesis, it is possible that Sertoli cells or other testicular cell populations also may contribute to MEHP effects.

## Data Availability

The raw data supporting the conclusions of this article will be made available by the authors, without undue reservation.
